# Independent Association between Epicardial Adipose Tissue Volume and Recurrence of Idiopathic Ventricular Tachycardia after Ablation

**DOI:** 10.31083/j.rcm2407189

**Published:** 2023-06-30

**Authors:** Zhe Wang, Yijia Wang, Jiawei Chen, Hehe Guo, Lichen Ren, Xiaojie Chen, Yingwei Chen, Yihong Sun

**Affiliations:** ^1^Department of Cardiology, China-Japan Friendship Hospital (Institute of Clinical Medical Sciences), Chinese Academy of Medical Sciences & Peking Union Medical College, 100029 Beijing, China; ^2^Department of Cardiology, Beijing Hospital, Chinese Academy of Medical Sciences & Peking Union Medical College, 100029 Beijing, China; ^3^Department of Cardiology, The First Affiliated Hospital of Zhengzhou University, 450052 Zhengzhou, Henan, China; ^4^Department of Radiology, The First Affiliated Hospital of Zhengzhou University, 450052 Zhengzhou, Henan, China; ^5^Department of Cardiology, China-Japan Friendship Hospital, 100029 Beijing, China

**Keywords:** idiopathic ventricular tachycardia, epicardial adipose tissue, computed tomography, radiofrequency ablation, recurrence

## Abstract

**Background:**

Epicardial adipose tissue (EAT) thickness is an independent predictor for the recurrence of premature ventricular beats after ablation. However, it is unclear whether EAT volume is associated with the recurrence of idiopathic ventricular tachycardia (IVT) following ablation. This study sought to investigate the association between EAT volume and IVT recurrence following radiofrequency ablation for IVT patients.

**Methods:**

This retrospective study included 69 IVT patients undergoing computed tomography examination before ablation who underwent their first catheter ablation between 2017 and 2021. The predictive value of EAT volume for IVT recurrence following ablation was assessed.

**Results:**

During the follow-up period (median: 540 days; range: 253–929 days), 26.1% (18/69) of the patients experienced IVT recurrence. The cut-off point of EAT volume for predicting IVT recurrence was 160.30 mL, and the area under the curve (AUC) was 0.751 (95% confidence interval (CI): 0.615–0.887) by the receiver operating characteristic curve. Kaplan-Meier analysis showed that patients with larger EAT volumes had higher cumulative rates of IVT recurrence. Multivariable analysis also revealed that EAT volume (per 10 mL increase; hazard ratio (HR): 1.16, 95% CI: 1.03–1.32, *p* = 0.018) was independently associated with IVT recurrence. Furthermore, patients with an epicardial site of IVT had a significantly larger EAT volume than IVT patients with non-epicardial origins.

**Conclusions:**

A larger EAT volume may be associated with IVT recurrence after catheter ablation. EAT volume may be helpful for risk stratification in patients undergoing IVT ablation.

## 1. Introduction

Catheter ablation is particularly suitable for patients with idiopathic ventricular tachycardia (IVT) without structural heart disease [1]. IVT ablation has increasingly been used as a major therapy, and can significantly improve a patient’s quality of life [2, 3]. However, patients with IVT ablation still have high recurrence rates. A previous study reported a 38.0% recurrence of IVT following ablation in patients with a structurally normal heart during a median 572-day follow-up period [4]. Most patients with IVT have risk factors and it is important to identify risk factors that increase the risk of recurrence of ablation procedures [5]. However, the risk factors related to the recurrence risk of IVT have not been fully described [6].

Epicardial adipose tissue (EAT), a unique visceral adipose tissue, is located between the myocardium and visceral pericardium without an intervening fascial plane [7]. A study found that patients with frequent ventricular premature beats had increased EAT thickness compared to control patients [8]. Another study revealed that ventricular tachycardia (VT) frequently occurs in fatty infiltrated myocardium or EAT-rich patients, suggesting that EAT potentially plays an important role in promoting arrhythmogenesis [9]. At present, the relationship between EAT volume and IVT following ablation is unclear. This study sought to investigate the relationship between post-ablation IVT recurrence and pre-procedural EAT volume using non-contrast computed tomography (CT).

## 2. Methods

### 2.1 Study Design and Population

This retrospective study included patients with IVT who underwent radiofrequency catheter ablation (RFCA) between January 2017 and September 2021 at the First Affiliated Hospital of Zhengzhou University. The inclusion criteria were: (1) IVT patients who underwent non-contrast CT before ablation; (2) absence of structural heart disease. Patients with a history of prior ablation, acute IVT ablation failure, or poor/insufficient CT images were excluded. Patients who died or were lost to follow-up were also excluded from the analysis. Patients with a diagnosis of IVT were identified using the international classification of diseases (ICD) code in our hospital’s electronic health record systems. IVT diagnosis in our study was defined as an absence of structural heart disease and ventricular tachycardia lasting ≥30 s. The study complied with the Declaration of Helsinki. The study protocol was authorized by the local institution’s ethics committee (2022-KY-043).

### 2.2 Clinical and Laboratory Data

The following study data were collected from all patients: demographic parameters, comorbidities, echocardiographic parameters (left ventricular ejection fraction, left atrial [LA] diameter, left ventricular end-diastolic diameter, E/A ratios), CT parameters (EAT volume and attenuation), and medications on admission.

### 2.3 CT Acquisition

All patients used a dual-source CT system (Somatom Force, Siemens Healthineers, Germany) in one session without changing their position. Non-contrast CT was performed at 120 kV. The tube current was adjusted to the body’s habitus. The images were reconstructed with a slice thickness of 0.5 mm, a reconstruction increment of 0.5 mm with a medium soft-tissue convolution kernel (B26F), and a reconstructed matrix size of 512 × 512. Each reconstructed image was transferred to the reconstructed workstation for post-processing.

### 2.4 EAT Assessment Using Non-Contrast CT

**Fig. 1.**
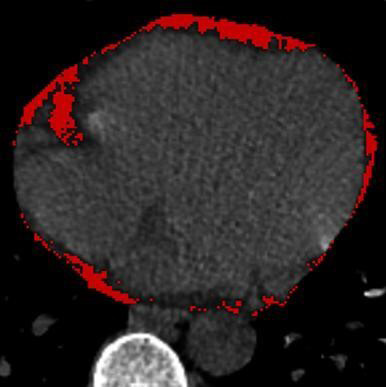
**The measurement of EAT volume by non-contrast CT**. Abbreviations: CT, computed tomography; EAT, epicardial adipose tissue.

EAT is a low-density margin that encases the myocardium in the pericardial space on CT. EAT volume was defined as a non-contrast CT density ranging from –195 to 45 Hounsfield units (HU) [10, 11]. EAT volume and attenuation were quantified using dedicated semiautomatic software (Syngo via Frontier Cardiac Risk Assessment, version 1.2.3, Siemens Healthineers, Germany), as shown in Fig. 1. The software automatically delineated and identified EAT and manually adjusted the contour of the EAT volume, if necessary. Two experienced radiologists quantified the image, and operators were blinded to the participant’s clinical data.

### 2.5 Ablation Protocol 

Antiarrhythmic medications were stopped for a period of five half-lives before ablation whenever possible [12]. The ablation procedure was performed when the patient was awake condition, except for 4 patients with pain intolerance who were administered conscious sedation with remifentanil. A three-dimensional electroanatomic mapping system (CARTO system, Johnson & Johnson Medical, Biosense Webster Inc., Diamond Bar, CA, USA) was used during the ablation procedure. IVT spontaneously occurred or induced using programmed stimulation. Programmed electric stimulation from the right ventricular apex and outflow tract and up to three extrastimuli were used until a ventricular effective refractory period, or coupling interval of 200 ms, was reached. If the IVT was non-inducible at baseline, the stimulation protocol was repeated after isoprenaline infusion. Isoprenaline was administered at a lower dose and was gradually increased. Activation mapping was conducted to investigate ablation targets. The beginning of the surface electrocardiography (ECG) $V_{1}$ at the IVT was taken as a reference. The IVT map was acquired to identify the earliest ventricular activation potential in patients with inducible, stable, and hemodynamically tolerated IVT.

The earliest activation site was mapped using an irrigated-tip mapping catheter (ThermoCool, Biosense Webster, Inc, Irvine, CA, USA). The irrigation rate was 17 mL/min with a power of 30–40 W on the endocardial sides ablation and 15–30 W on the epicardial side ablation. Radiofrequency energy applied during IVT was used for 30–60 s. When IVT was terminated during energy application, the application was continued for 120–200 s at the site. Furthermore, epicardial ablation was performed when the clinical or induced IVT suggested an epicardial origin and endocardial ablation was unsuccessful. If mapping demonstrated a suitable ablation site within the coronary venous, ablation was attempted within the coronary vein. If ablation sites were near the coronary arteries, coronary angiography was performed. RFCA energy should not be applied within 5 mm of the coronary artery to avoid arterial injury. Successful IVT elimination may need energy delivery from adjacent locations [13]. If ablation at the earliest endocardial site and coronary sinus ablation were ineffective or transiently effective, a subxiphoid puncture was performed for subsequent ablation [14]. Acute procedural success was defined as the elimination of all sustained inducible IVT. VT could not be induced despite programmed electrical stimulation with isoproterenol infusion after 30 min of observation.

### 2.6 Outcomes and Follow-Up 

Patients were routinely evaluated 1 month after ablation and then at 3–6-month intervals. Follow-up visits included 12-lead ECG, 24-h Holter monitoring, and clinical assessment. Patients with symptoms related to IVT were asked to immediately complete an additional outpatient visit. If patients were lost during the follow-up period, they were contacted over the telephone to determine whether IVT had recurred and encouraged to resume their follow-up visits. IVT episodes were defined as ventricular tachycardia lasting ≥30 s or requiring appropriate intervention for termination. The study’s endpoint was IVT recurrence after ablation. All patients with successful procedures were eligible to discontinue antiarrhythmic drugs after RFCA. If a recurrence occurred after ablation, the patient’s antiarrhythmic drugs were reinstituted.

### 2.7 Statistical Analysis

Categorical variables are presented as numbers (percentages) and were compared between the two groups using the Pearson chi-squared or Fisher’s exact tests. The values of the continuous variables were described as the mean ± standard deviation or median (Q1, Q3 quartiles) compared among groups using Student’s *t*-test or Mann-Whitney U test, depending on whether the data were normally distributed. The Pearson correlation test was used to determine the association between age and body mass index (BMI) with EAT volume. Receiver operating characteristic curve analysis was performed to identify the discriminative power, specificity, and sensitivity for predicting the recurrence of IVT ablation based on the pre-procedural EAT volume. Multivariable Cox regression analysis was used to investigate risk factors for post-ablation recurrence, adjusted for other variables. The Kaplan-Meier curve was constructed to investigate freedom from recurrent IVT. The *p*-value is the result of two-tailed tests. All statistical analyses were performed using SPSS (version 21.0; SPSS Inc., Chicago, IL, USA) and R language version 4.0.3 (R Foundation for Statistical Computing, Vienna, Austria).

## 3. Results

### 3.1 Patients’ Characteristics 

A total of 101 patients with IVT were screened for eligibility. Six-nine patients were included in the final analysis (**Supplementary Fig. 1**). The time from non-contrast CT to RFCA was 2 (1, 3) days. The mean age was 41.7 (SD: 16.7) years old. 43.5% (30/69) of IVT patients who underwent cardiac magnetic resonance (CMR) without undetected cardiomyopathy.

The median follow-up time was 540 (range: 253–929) days. 26.1% (18/69) of IVT patients had recurrent IVT. All patients were divided into two groups according to whether they had an IVT recurrence. Age and BMI were not significantly different between the two groups. Patients with IVT recurrence were more likely to have a larger LA diameter and EAT volume than those without recurrent IVT.

### 3.2 Procedure-Related Characteristics

**Fig. 2.**
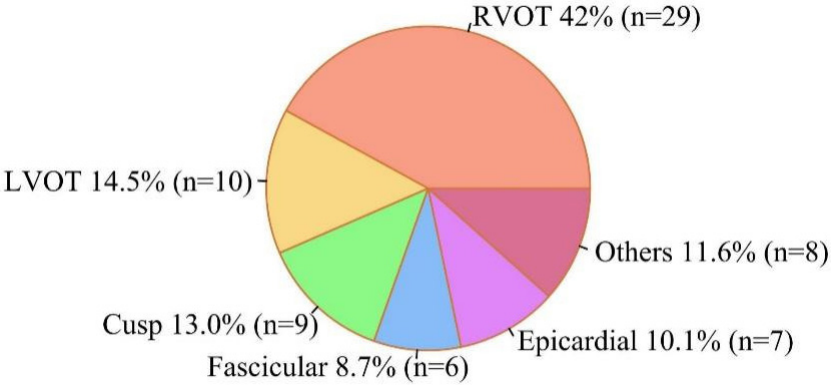
**Distribution of IVT origin locations**. Abbreviations: LVOT, left ventricular outflow tract; IVT, idiopathic ventricular tachycardia; RVOT, right ventricular outflow tract.

Procedure-related complications were low in all patients and included two patients with vascular access complications and one with a pericardial effusion. There was no significant difference in total procedure time between the two groups. Compared to the non-recurrence group, the recurrence group had a non-statistically lower proportion of IVT which originated from the RVOT locations, as shown in Table 1. Of the 69 patients with IVT, 29 (42.0%), 10 (14.5%), 6 (8.7%), 7 (10.1%), 9 (13.0%), and 8 (11.6%) patients had IVT originating from the right ventricular outflow tract (RVOT), left ventricular outflow tract (LVOT), fascicular, epicardial, cusp, and other sites, respectively, as described in Fig. 2. The highest rate of IVT recurrence was in patients with an epicardial origin (42.9%). The lowest recurrence rate was in patients with a ROVT origin (13.8%), as described in **Supplementary Fig. 2**.

**Table 1.** **Baseline characteristics of patients according to post-ablation IVT recurrence**.

Variable		All	Non-recurrence	Recurrence	*p*-value
Patients		69	51	18	
Age, years		41.7 ± 16.7	39.4 ± 16.0	48.2 ± 17.6	0.054
Female gender		25 (36.2%)	20 (39.2%)	5 (27.8%)	0.385
BMI, kg/$m^{2}$		24.9 ± 3.3	24.7 ± 2.9	25.6 ± 4.4	0.305
Hypertension		14 (20.3%)	9 (17.6%)	5 (27.8%)	0.358
Diabetes mellitus		5 (7.2%)	4 (7.8%)	1 (5.6%)	1.000
Medication on admission					
	ACEI/ARB	9 (13.0%)	5 (9.8%)	4 (22.2%)	0.226
	Amiodarone	18 (26.1%)	12 (23.5%)	6 (33.3%)	0.319
	Beta-blocker	22 (31.9%)	18 (35.3%)	4 (22.2%)	0.551
	CCB	13 (18.8%)	9 (17.6%)	4 (22.2%)	0.730
	Statins	6 (8.7%)	4 (7.8%)	2 (11.1%)	0.631
Laboratory test					
	WBC, mmol/L	7.4 ± 2.2	7.3 ± 2.0	7.8 ± 2.9	0.406
	HS-CRP (>2 mg/L)	22 (31.9%)	16 (31.4%)	6 (33.3%)	0.878
Echocardiographic variables					
	LVEF, %	59.5 ± 8.8	60.4 ± 7.5	57.0 ± 11.7	0.157
	LA diameter, mm	34.1 ± 5.7	33.2 ± 4.7	36.6 ± 7.7	0.033
	E/A ratios >1	36 (52.2%)	28 (54.9%)	8 (44.4%)	0.422
CT variables					
	EAT volume, mL	146.7 ± 66.8	129.7 ± 53.1	194.9 ± 78.9	0.004
	EAT attenuation, HU	–89.5 ± 7.4	–89.8 ± 6.7	–88.7 ± 9.2	0.577
Procedural characteristics					
	Total procedure time (min)	156 (145, 193)	156 (145, 193)	164 (151.5, 202.5)	0.118
	Origin RVOT locations	29 (42.0%)	25 (49.0%)	4 (22.2%)	0.057

Note: Continuous data are presented as means ± standard deviation (SD) or median (inter-quartile range), and categorical data were shown as n (%). Abbreviations: ACEI, angiotensin-converting enzyme inhibitor; ARB, angiotensin receptor blocker; BMI, body mass index; CCB, calcium channel blocker; CT, computed tomography; EAT, epicardial adipose tissue; HS-CRP, high-sensitivity C-reactive protein; HU, Hounsfield units; LA, left atrial; LVEF, left ventricular ejection fraction; RVOT, right ventricular outflow tract; WBC, white blood cell; IVT, idiopathic ventricular tachycardia.

### 3.3 EAT Volume Characteristics

**Fig. 3.**
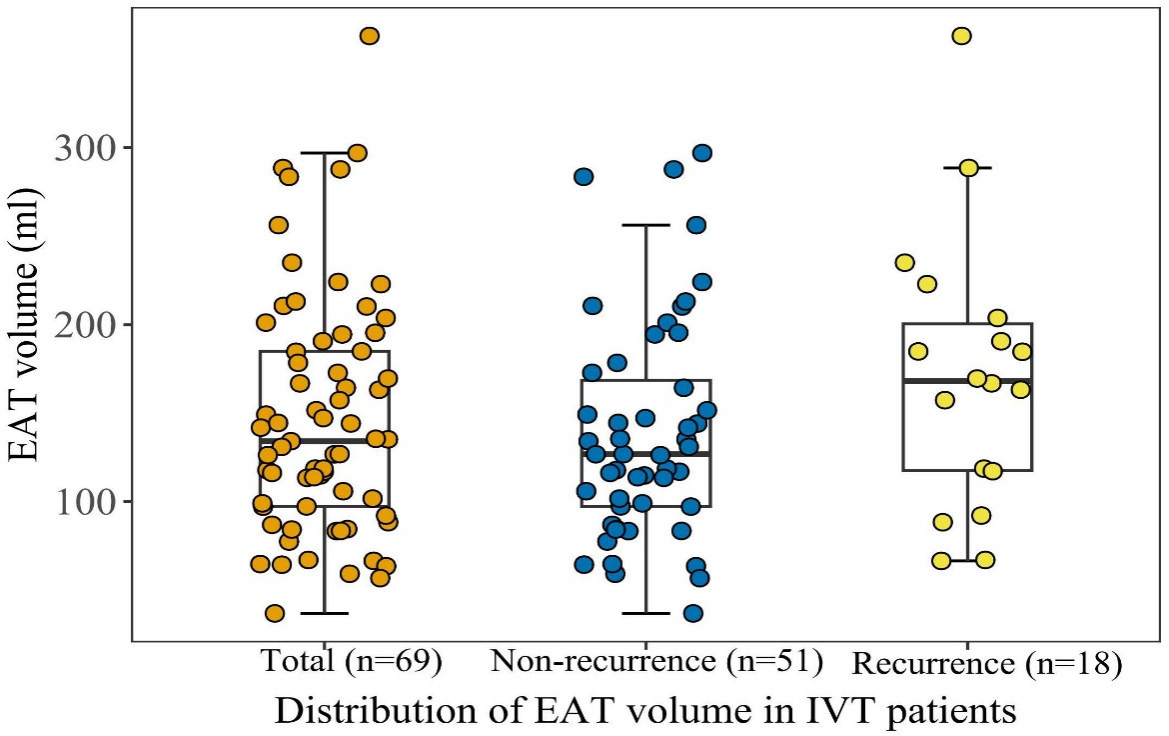
**Distribution of EAT volume in IVT patients according to post-ablation recurrence**. Different color dots represent measured epicardial fat volume; top of the box, 75th percentile; horizontal line, 50th percentile (median); bottom of the box, 25th percentile; whiskers, maximum and minimum EAT volume except for outliers, respectively. Abbreviations: EAT, epicardial adipose tissue; IVT, idiopathic ventricular tachycardia.

The volume of EAT distribution is shown in Fig. 3. The mean value of EAT was 146.7 (SD: 66.8) mL. Patients with IVT recurrence had larger EAT volumes than patients without recurrence. The cut-off of EAT volume for the prediction of IVT recurrence was 160.30 mL. The area under curve (AUC) was 0.751 (95% confidence interval (CI): 0.615–0.887), and the specificity and sensitivity were 76.5% and 72.2%, respectively (**Supplementary Fig. 3**). Furthermore, EAT volume was significantly correlated with age (r = 0.388, *p* = 0.001) and BMI (r = 0.450, *p* < 0.001), (**Supplementary Figs. 4,5**).

### 3.4 Predictive Value of EAT Volume

**Fig. 4.**
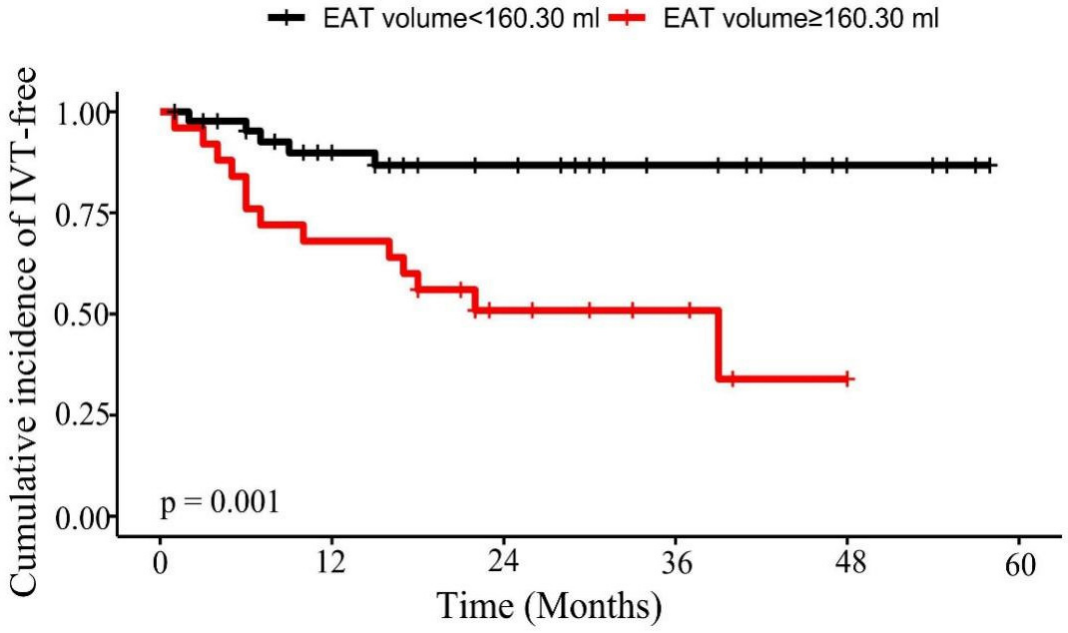
**Kaplan-Meier curve for IVT recurrence following ablation**. EAT volume as a categorical variable was used to predict IVT recurrence according to the cut-off values (160.30 mL). Abbreviations: EAT, epicardial adipose tissue; IVT, idiopathic ventricular tachycardia.

Variables for which univariable analysis showed a *p*-value < 0.10 were included in the multivariable Cox analysis, as depicted in Table 2. Multivariable analysis revealed that EAT volume (per 10 mL increase; hazard ratio (HR): 1.16, 95% CI: 1.03–1.32, *p* = 0.018) was independently associated with post-ablation IVT recurrence after adjusting for other factors. The Kaplan-Meier curves for freedom from IVT recurrence after ablation according to the cut-off value of EAT volume (160.30 mL) are shown in Fig. 4.

**Table 2.** **Risk factors for recurrence of IVT by multivariate Cox regression analysis model**.

Variable	HR (95% CI)	*p*-value
Age	1.02 (0.98–1.06)	0.363
Origin of RVOT sites	1.29 (0.30–5.52)	0.730
LA diameter	0.97 (0.85–1.11)	0.685
EAT volume, per increase 10 mL	1.16 (1.03–1.32)	0.018

Abbreviations: CI, confidence interval; EAT, epicardial adipose tissue; HR, hazard ratio; LA, left atrial; RVOT, right ventricular outflow tract; IVT, idiopathic ventricular tachycardia.

### 3.5 Further EAT Analysis 

**Fig. 5.**
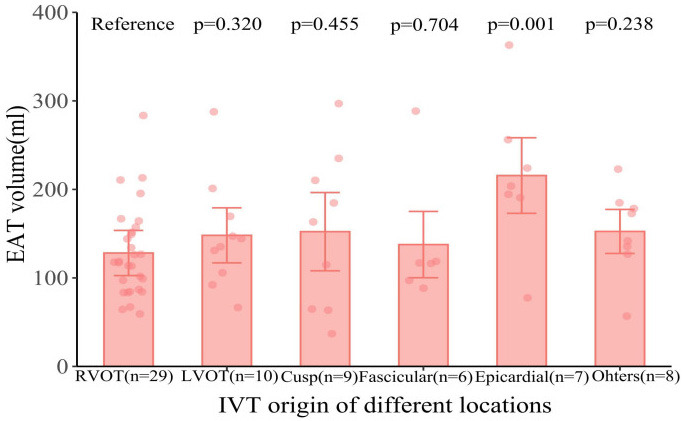
**Comparison of EAT volume of IVT patients according to locations of origin**. Notes: The dots represent measured EAT volume. Abbreviations: EAT, epicardial adipose tissue; IVT, idiopathic ventricular tachycardia; LVOT, left ventricular outflow tract; RVOT, right ventricular outflow tract.

EAT volume was significantly larger in patients with IVT originating from the epicardial location than in IVT patients with originating from the RVOT location. However, EAT volume in IVT patients originating from the LVOT, fascicular, epicardial, cusp, and other sites was not statistically larger than that of IVT patients originating from the RVOT, as described in Fig. 5.

## 4. Discussion

To the best of our knowledge, this study is the first to analyse the clinical characteristics of EAT volume using non-contrast CT in IVT patients following radiofrequency ablation. We found that EAT volume was independently associated with IVT recurrence. A larger EAT volume was observed in IVT patients who originated from epicardial locations. EAT volume, as a quantitative measure, maybe a powerful potential diagnostic tool for the risk of IVT recurrence.

### 4.1 EAT and Arrhythmias

An increasing number of studies are investigating the association between EAT and cardiovascular diseases [15]. EAT accumulation is related to myocardial ischemia and obstructive coronary heart disease [16]. Some studies have revealed that a high EAT volume has a strong association with the risk of atrial fibrillation (AF) [17]. The comparison between patients with AF and healthy participants resulted in a 32.0 mL difference in EAT volume, indicating that patients with AF have higher EAT volume [18]. EAT volume is an independent predictor of AF recurrence following ablation [18]. Pericardial fat is strongly associated with ventricular arrhythmia development in patients with heart failure [19]. A previous study demonstrated that patients with recurrence after ablation have a larger right and left atrioventricular EAT thickness than those with non-recurrence in 61 patients with VT [9]. Our study enrolled IVT patients with non-structural heart disease, which differed from those of previous studies. VT ablation is feasible for most patients and the risk of complications is low [14]. We found that a large EAT volume was independently associated with IVT recurrence following ablation. Additionally, our study included 6 cases (8.7%) with fascicular IVT of origin. Fascicular IVT has a different pathophysiology compared to other IVT sites [20]. A higher recurrence rate of 33.3% (2/6) in patients with fascicular IVT than in the previous literature, possibly due to bias caused by the small sample size [21]. EAT volume may be used as a non-invasive index to predict postoperative recurrence in patients with IVT.

### 4.2 Clinical Characteristics of EAT 

Age and BMI were associated with EAT volume in our study, which is consistent with a previous study [22]. Fatty deposits are exceedingly prevalent within the scar and commonly extend to the sub-endocardium with varying levels of transmural penetration [23]. Epicardial ablation has risen to an alternative part of the treatment of VT [14]. We found that IVT patients with originating from epicardial locations had a high recurrence rate, which is consistent with a previous study [24]. Our study revealed that IVT patients originating from an epicardial location had a larger EAT volume than those originating from RVOT locations. EAT thickness >7 mm is a prominent factor for epicardial ablation failure in ventricular arrhythmias patients [25]. This discrepancy may be because radiofrequency ablation is less likely to produce effective ablation lesions where there is an increased EAT accumulation. The accumulated EAT could reduce the electrogram amplitude and cause these areas to be mistaken for scar tissue [26]. Hence, EAT may alter local electrophysiology, and help identify higher-risk candidates [27]. Future studies are needed to confirm the correlation between EAT and IVT.

### 4.3 Potential Mechanisms Linking Increased EAT to IVT

The mechanism underlying increased EAT with IVT recurrence remains unclear. Several key mechanisms may explain these findings. First, the increased EAT is associated with conduction delay and interatrial block [28]. An increased EAT is related to a widened and fragmented QRS, which indicates slow ventricular conduction or hypertrophy [29]. EAT may contribute to the development of IVT by triggering an increase in activity and re-entry mechanisms [9]. Second, EAT could secrete various substances that affect the electrophysiology of cardiomyocytes by modulating ion currents, or electrical coupling [30]. Excessive EAT-derived fatty acids can be taken up by cardiomyocytes and lead to ectopic myocardial lipid accumulation [31]. Adipocytes of EAT can secrete adipokines, and adipokine infiltration can cause electrical remodeling of cardiomyocytes and promote myocardial fibrosis [32]. The infarcted myocardium can also be infiltrated by lipoma metaplasia, which may increase scar formation [33]. Furthermore, EAT is a transmitter of the deleterious effects of inflammation and metabolic disturbances on the cardiac [27]. The increased size and number of adipocytes in the accumulated EAT, may ultimately lead to increased secretion of pro-inflammatory cytokines and down-regulation of the secretion of anti-inflammatory factors [34]. High levels of inflammatory cytokines could lead to cardiac remodeling and induce the development of arrhythmias [35]. In summary, the structural and paracrine crosstalk between EAT and cardiomyocytes promotes IVT.

### 4.4 CT Analysis

Previous studies have shown that non-contrast CT can be used to quantify EAT volume, avoiding the necessity of complex ECG-contrast acquisition. EAT volume by non-contrast CT measurements has consistently and reliably been correlated with contrast coronary CT angiography [10, 36]. Non-contrast CT has the advantages of convenience and reduced radiation dose and intensity, which is more applicable to the general population [11].

## 5. Limitations

This study had some limitations. First, this was an observational, retrospective study with a small sample size, which might bias the results. 56.5% (39/69) IVT patients did not have a preoperative CMR, which may have missed a few patients with cardiomyopathy. Meanwhile, we did not analyze the correlation between the number of IVT occurring preoperatively and EAT volume. However, the study follow-up was prospective and detailed, which improved the quality of the data. Second, the patients in this study were primarily from tertiary referral centers, which may not reflect the general population. Experienced electrophysiologists performed all the procedures, and the results were obtained from experienced centers. Additionally, the study excluded data on cases of failed immediate catheter ablation and structural heart disease, which may limit the generalizability of the results [37]. We were unable to assess the potential association between EAT and scarred areas. Further studies using more detailed EAT imaging (such as CMR, and intracardiac echocardiography) may be needed [38]. Finally, the study used non-contrast CT examinations with some degradation in image quality.

## 6. Conclusions

EAT volume was larger in patients with IVT recurrence after RFCA than in those without recurrence. IVT patients originating from an epicardial location had larger EAT volumes. EAT volume may help to identify risk stratification in IVT patients following radiofrequency ablation.

## Data Availability

The data supporting the findings of this study are available on request.

## Supplementary Material

Supplementary material associated with this article can be found, in the online version, at https://doi.org/10.31083/j.rcm2407189.
