# Supplementary material for: Independent Association between Epicardial Adipose Tissue Volume and Recurrence of Idiopathic Ventricular Tachycardia after Ablation
Source: Rev Cardiovasc Med. 2023 Jun 30;24(7):189. doi: 10.31083/j.rcm2407189 (PMC11266454; doi:10.31083/j.rcm2407189)
Supplement: Supplementary file 1 [file 2153-8174-24-7-189-s1.docx]

**
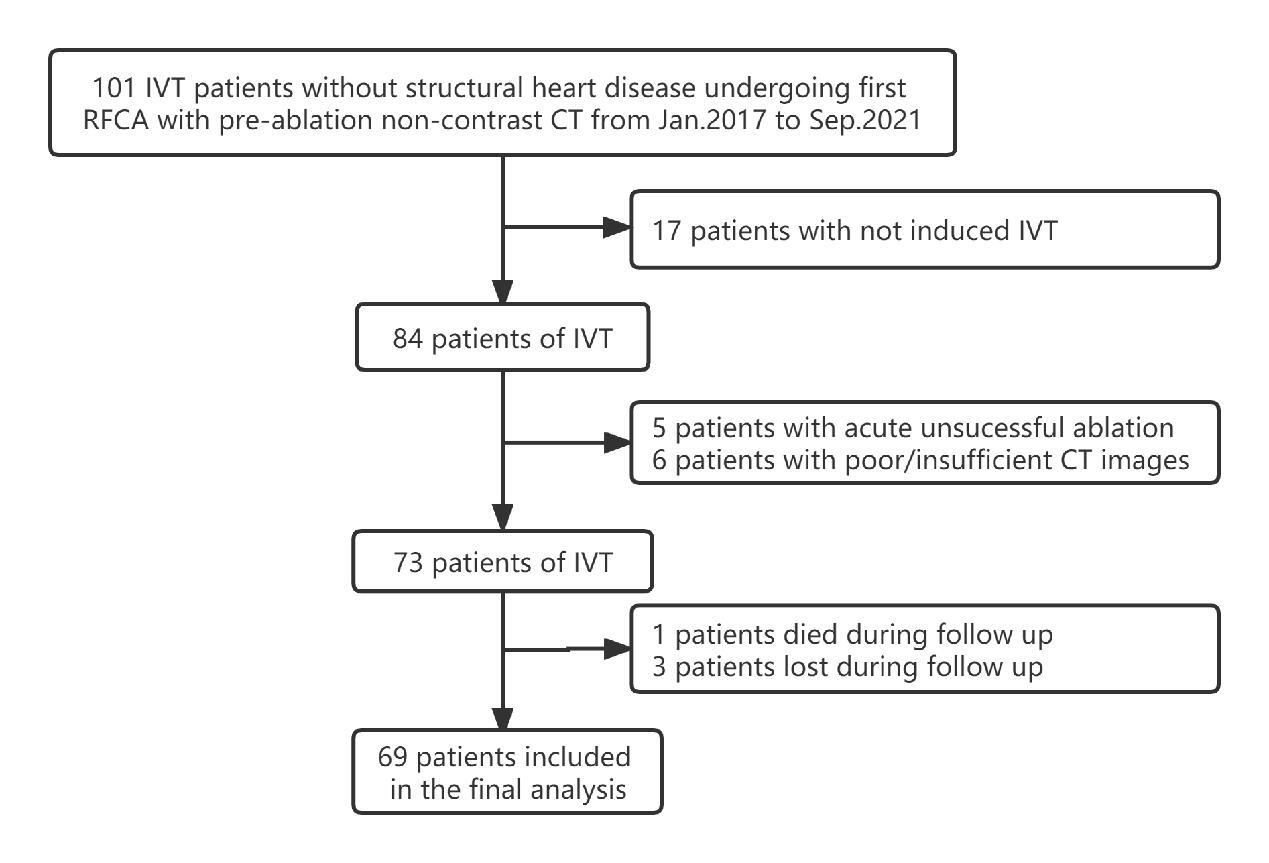
**

**Supplementary Fig. 1. The flowchart of the study cohort.** Abbreviations: CT, computed tomography; IVT, idiopathic ventricular tachycardia; RFCA, radiofrequency catheter ablation.


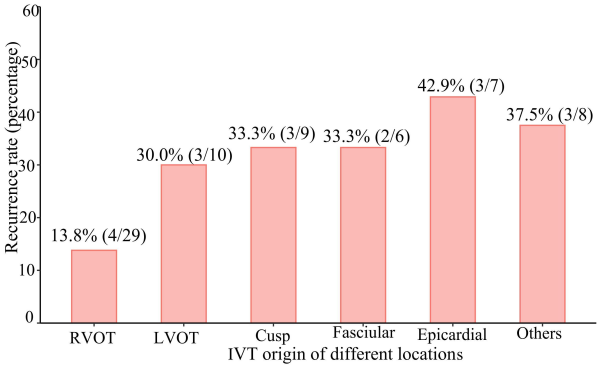


**Supplementary Fig. 2. Recurrence rates and IVT sites.** Abbreviations: IVT, idiopathic ventricular tachycardia; LVOT, left ventricular outflow tract; RVOT, right ventricular outflow tract.


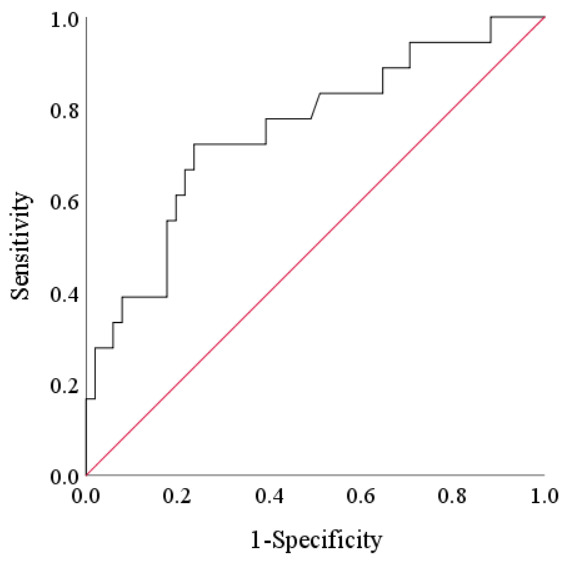


**Supplementary Fig. 3.** **Receiver operating characteristic curve illustrating the accuracy of EAT volume for predicting post-ablation IVT recurrence.** Abbreviations: EAT, epicardial adipose tissue; IVT, idiopathic ventricular tachycardia.


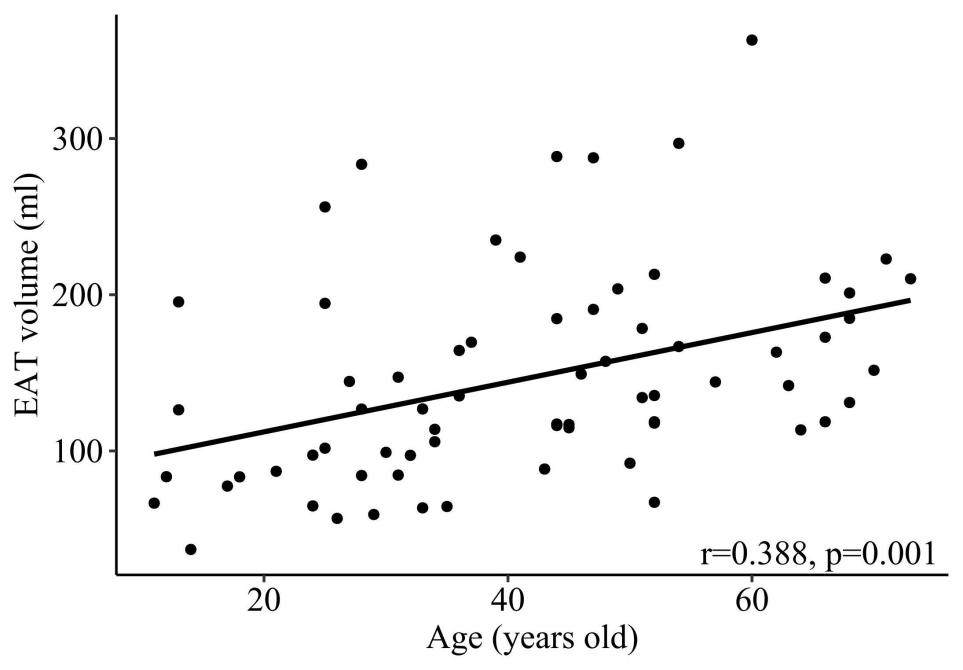


**Supplementary Fig. 4.** **The association between EAT volume and age.** Abbreviations: EAT, epicardial adipose tissue.


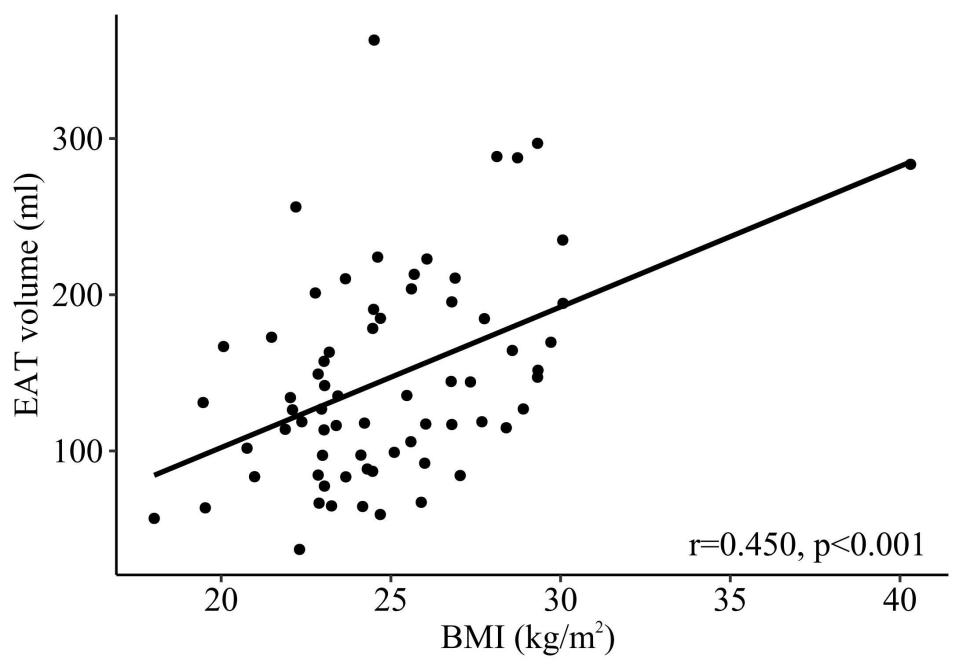


**Supplementary Fig. 5.** **The association between EAT volume and BMI.**  Abbreviations: BMI, body weight index; EAT, epicardial adipose tissue.
